# Examining the development and utilisation of Community-Based Health Information Systems (CBHIS) in Africa: A Scoping Review

**DOI:** 10.12688/wellcomeopenres.22780.2

**Published:** 2024-09-24

**Authors:** Beatrice Kuvuna, Moriasi Nyanchoka, Fatuma Guleid, Michael Ogutu, Benjamin Tsofa, Jacinta Nzinga

**Affiliations:** 1 Health Economics Research Unit, KEMRI-Wellcome Trust Research Programme, Nairobi, Kenya; 2 Health Systems and Research Ethics Department, KEMRI-Wellcome Trust Research Programme, Kilifi, Kenya; 3 Liverpool School of Tropical Medicine, Liverpool, England, UK

**Keywords:** Community-based health information systems (CBHIS), Community health systems (CHS), Health systems, Data utilisation, data-driven decision-making, Community accessibility, Community empowerment, Africa

## Abstract

**Introduction:**

The community-based health information system (CBHIS) is a vital component of the community health system, as it assesses community-level healthcare service delivery and generates data for community health programme planning, monitoring, and evaluation. CBHIS promotes data-driven decision-making, by identifying priority interventions and programs, guiding resource allocation, and contributing to evidence-based policy development.

**Objective:**

This scoping review aims to comprehensively examine the use of CBHIS in African countries, focusing on data generation, pathways, utilization of CBHIS data, community accessibility to the data and use of the data to empower communities.

**Methods:**

We utilised Arksey and O'Malley's scoping review methodology. We searched eight databases: PubMed, EMBASE, HINARI, Cochrane Library, Web of Science, Scopus, Google Scholar, and grey literature databases (Open Grey and OAIster). We synthesized findings using a thematic approach.

**Results:**

Our review included 55 articles from 27 African countries, primarily in Eastern and Southern Africa, followed by West Africa. Most of the studies were either quantitative (42%) or qualitative (33%). Paper-based systems are primarily used for data collection in most countries, but some have adopted electronic/mobile-based systems or both. The data flow for CBHIS varies by country and the tools used for data collection. CBHIS data informs policies, resource allocation, staffing, community health dialogues, and commodity supplies for community health programmes. Community dialogue is the most common approach for community engagement, empowerment, and sharing of CBHIS data with communities. Community empowerment tends towards health promotion activities and health provider-led approaches.

**Conclusion:**

CBHIS utilizes both paper-based and electronic-based systems to collect and process data. Nevertheless, most countries rely on paper-based systems. Most of the CBHIS investments have focused on its digitization and enhancing data collection, process, and quality. However, there is a need to shift the emphasis towards enabling data utilisation at the community level and community empowerment.

## Plain Language Summary

For community health services and systems to work well, health managers and other data users, including policy and decision-makers, need a community-based health information system (CBHIS) that produces reliable and timely information on how well these services are working and that supports the use of CBHIS data to improve community health service delivery. This scoping review aimed to explore the use of CBHIS in African countries. It focused on data generation, pathways, use of CBHIS data, community data access, and use of CBHIS data to empower communities. The review authors collected and analysed all relevant studies to answer this question and found 55 articles from 27 African countries. The review found that most countries use paper-based information systems for data collection, while some have adopted electronic and digital systems. CBHIS also collects information on human resources, medicines, and supply systems. CBHIS data are used to guide policy development, allocate resources, track commodities supplies, staff for community health programmes and organise community health dialogues. Community dialogue is the most common approach for engaging, empowering, and sharing CBHIS data with communities. Community empowerment involves activities that promote health and health provider-led approaches. There is a need to focus on enabling the use of data at the community level and empowerment.

## Introduction

Community Health Systems (CHS), defined as the interface between community systems and the formal health system, is the most accessible, equitable, cost-effective, and efficient approach to improving access and coverage of health services in a continuum of the primary health care (PHC) system ^1^. A strong CHS is critical for delivering accessible, quality, cost-effective preventive and treatment services, including emergency care ^2^.

The Astana Declaration on PHC in 2018 fostered a renewed global interest in strengthening CHS in the context of the Universal Health Coverage (UHC) and other Sustainable Development Goals (SDGs). Integrating community health approaches in health systems is now considered paramount ^3^, as CHS can help monitor population-level health system performance, track key indicators related to UHC and other health-related SDGs, and enhance the quality of health information ^4^. The success of the CHS in handling global crises, such as the Ebola epidemic in West Africa and the COVID-19 pandemic, further emphasizes its importance in providing essential health services at the community level and supporting public health emergency preparedness and response ^2^. CHS is thus seen as a crucial aspect of PHC, and its strengthening is essential for achieving UHC and other health-related SDGs ^5^.

A community-based health information system (CBHIS) is a vital system that encompasses information about the collection and flow of data, assessment and enhancement of data quality, and utilization of community health data. It is essential for ensuring accurate data collection to support governance and management of CHS and decision-making at local, sub-national, and national levels ^4, 6, 7^. CBHIS data also enable advocacy for vulnerable populations ^6^, serve as an early warning alert and response (EWAR) tool, support case management and community health units/posts, enable health trend analyses, and reinforce the communication of health challenges to diverse groups ^8^.

The four fundamental functions of CBHISs are data generation, data compilation, analysis and synthesis, and communication and use ^8^. CBHISs gather health and other relevant data, ensure its quality, relevance, and timeliness, and transform it into useful information for health-related decision-making. However, the CBHIS requires critical health system inputs, including human resources (community health workers), budgetary allocation, and day-to-day operational management, to function efficiently ^4, 5, 8, 9^. Many low- and middle-income countries (LMICs) face challenges in establishing and maintaining CBHIS due to insufficient government funding ^4^, leading to significant gaps in community-level health data quality ^5, 6, 10^, and thus limiting the demand and utilisation of CBHIS in decision-making processes ^11^. This underutilization of CBHIS data in decision-making processes can be attributed to fragmented community-based reporting systems ^10^, lack of coordination between data producers and users ^12, 13^, multiple parallel information subsystems ^13^, and variations in the decentralization of community health decisions ^14^. Furthermore, limited integration of CBHIS with the formal Health Management Information System (HMIS), insufficient funding for the CHS ^2, 4, 6, 15^, and contextual factors beyond technical aspects of data processes and organizational aspects impact the use of evidence in the CHS ^13, 14^.

Although several African countries have embraced digital platforms, most countries (71 %) continue to rely on paper-based systems to collect CBHIS data ^1, 2^. Several infrastructural constraints, including limited access to cell phones, stable electrical power supplies, and mobile networks, impede the adoption of digital systems ^10, 13, 16– 18^. However, some countries, such as Malawi, Zambia, Ghana, and Kenya, have successfully adopted simple feature phones with simple SMS-based reporting systems, enabling real-time data transmission to all healthcare systems ^4, 19^.

Several African countries have recently invested in enhancing their CHS and strengthening their CBHIS systems ^20^. These efforts have included the digitisation of existing CBHIS systems to improve community health programs and work towards providing universal access to PHC services ^21^. However, most CBHIS systems in these countries are partner-driven, program-specific, and heavily reliant on donors' and partners’ financial and technical support, as evidenced in the Democratic Republic of Congo (DRC), Egypt, Namibia, and Kenya ^1, 10, 17^. As a result, the landscape of CBHIS data is disjointed and fragmented, failing to integrate with the national HMIS ^10^.

There are limited reviews on CBHIS in Africa. A review by Mekonnen *et al*. ^4^ examined the current status and implementation challenges of CBHIS in LMICs-Africa but did not focus on CBHIS data processes, utilisation of CBHIS data on health system decision-making, or community access to CBHIS data and community empowerment. Our review focuses on these aspects of the CBHIS. We aim to address the gap in these aspects and inform efforts to enhance the CHS, ultimately contributing to improved community health service coverage and tracking progress towards UHC and other health-related SDGs.

## Methods

This scoping review adopted the Arksey and O'Malley’s Framework ^22^ to comprehensively examine the development, implementation, and utilization of CBHIS in Africa. This framework guided the methodological processes for our review. We adhered to the Preferred Reporting Items for Systematic Reviews and Meta-Analyses extension for Scoping Reviews (PRISMA-ScR) reporting guidelines ^23^. Our review was registered in the Open Science Framework ^24^.

### Eligibility criteria

We selected eligible studies using the Population, Concept, and Context (PCC) framework recommended for scoping reviews.

**Population:** We included primary studies of any study design that examined the CBHIS data sources, processes, pathways, utilisation, and accessibility of its data at the community level, involving community members, community health workers, local actors, and other stakeholders such as policymakers, community-based organizations, and health non-governmental organizations.

**Concept:** We included studies that explored and discussed various aspects of CBHIS, encompassing experiences in CBHIS development and utilisation, sources of CBHIS data, data generation processes, CBHIS data pathways, utilisation of CBHIS data in informing evidence-based decision-making, community accessibility of the CBHIS data and empowerment.

**Context/setting:** We included studies conducted in Africa.

We excluded studies on CBHIS conducted in high-income countries, studies published in languages other than English, reviews (systematic, scoping, literature, etc.), conference abstracts, opinions, and editorials on CBHIS.

### Information sources and search

We developed the search strategy in consultation with a health research librarian. An initial search was conducted in July 2023, and an updated search in November 2023. Seven databases were searched: PubMed, Embase, HINARI, Cochrane Library, Web of Science, Scopus, and Google Scholar. We also searched grey literature on open grey databases and hand-searched the references of included studies to identify additional literature. We limited our search to articles published in English between 2000 and 2023. The PubMed search strategy is presented in Additional File 1 (see Extended data, ^25^).

### Study selection

We exported references to the EndNoteX7 database, and duplicates were removed. Two independent reviewers performed study selection over two stages: title and abstract review and full-text review against the predefined eligible criteria, using Covidence. All disagreements were resolved by discussion or consulting with authorship team members for a consensus. Studies that met the inclusion criteria were selected for data extraction and charting.

### Data items and charting

A data extraction and charting form was developed and pilot-tested jointly with the research team to determine which variables to extract (Additional File 2) (see *Extended data*, ^25^). We extracted data on the following aspects: general study characteristics; sources of CBHIS data; data generation; pathways through which data is processed; utilisation of CBHIS data; and community accessibility to CBHIS data and empowerment. Data was extracted and exported from Covidence into Microsoft Excel software. One reviewer extracted data, and reviewers independently conducted quality checks of the extracted data. We resolved discrepancies by discussion between authors or consulting senior reviewers for a consensus.

### Synthesis of results

We synthesized the findings using a thematic approach commonly used in scoping reviews. We followed the PRISMA-ScR reporting guideline to present our findings.

## Results

### Selection of sources of evidence

Our search strategy yielded 7,101 records, of which 362 duplicates were excluded. We screened 6,762 titles and abstracts and excluded 6,498 articles. We screened 264 articles and included 55 articles in this review. The PRISMA flow diagram of the selection process and summary of the search results is provided in *Extended data,* ^25^.

### Characteristics of sources of evidence

We synthesized 55 studies from 27 African countries, primarily Eastern and Southern Africa, followed by the West African region. Of these, 52 were research studies, and only three were project/programme reports. Most studies were quantitative (42%), followed by qualitative studies (33%). Table 1 presents the characteristics of the studies, including country, study design, and topical focus. A summary of all key findings is provided in *Extended data,* ^25^.

**Table 1.** Characteristics of the included studies.

Category	Details	n (%)
Publication Type	Research Articles	52 (95%)
	Project/Programme Reports	3 (5%)
Year of publication	2007–2013	4 (7%)
	2014–2020	35 (64%)
	2021–2023	16 (29%)
Type of Study	Quantitative	23 (42%)
	Qualitative	18 (33%)
	Mixed Methods	4 (7%)
	Project report; Thesis; Project evaluation (3 each)	9 (16%)
	Workshop report	1 (2%)
	Not reported	1 (2%)
Study design	Cross-sectional	15 (27%)
	Qualitative	15 (27%)
	Project evaluation	5 (9%)
	Randomized controlled trial	5 (9%)
	Mixed methods	4 (7%)
	Case study	3 (5%)
	Cohort and Participatory action research (2 each)	4 (7%)
	Phenomenological; Secondary analysis; Assessment report (1 each)	3 (5%)
	Not reported	1 (2%)
Country	Kenya	13 (23%)
	Ethiopia	13 (23%)
	South Africa; Malawi (6 each)	12 (22%)
	Zambia	3 (5%)
	Multi-country (3): • Four countries: DRC, Egypt, Namibia, Mozambique • Seventeen West and Central African countries: Benin, Burkina Faso, Cameroon, Congo, DRC, Gambia, Ghana, Guinea Bissau, Ivory Coast, Liberia, Mali, Niger, Nigeria, Senegal, Sierra Leone, Chad, & Togo • Two countries: Kenya and Malawi	3 (5%)
	Mali, Ghana, Uganda (2 each)	6 (11%)
	Rwanda, Nigeria, Burkina Faso, Sierra Leone, Mozambique (1 each)	5 (9%)
Study setting	Health posts	11 (20%)
	Health facilities	7 (13%)
	Primary care sites/units	3 (5%)
	Community-based organizations (CBOs)	2 (4%)
	Health Centre	2 (4%)
	Health Office; National Health Insurance Pilot District	2 (4%)
	Not reported	28 (51%)
Summary of CBHIS *	Sources of CBHIS data	22 (40%)
	Processes in generating CBHIS data	51 (92%)
	CBHIS data pathways	25 (45%)
	Utilisation of CBHIS data	37 (67%)
	Community involvement and empowerment	17 (31%)

Note: *some studies report more than one detail

### Synthesis of results

***CBHIS data generation.*** CHWs are crucial for collecting CBHIS data. Included studies used various titles to describe CHWs based on their cadres and country of origin, including health extension workers (HEWs), community health volunteers (CHVs), community health extension workers (CHEWs), village pioneers, Health Surveillance Assistants (HSAs), community-based health workers (CBHWs), and village health teams (VHTs) ( Table 2). This paper uses CHWs as an all-encompassing term to cover all these designations for ease of reading and clarity. Table 2 summarises the CBHIS data collectors, standard data collection tools, and type of data collected.

**Table 2.** Summary of CBHIS data generation processes.

	Data collectors	Data collection tools/templates	Type of data collected at the community level	Data submission
Data generation processes	CHWs • Ethiopia (HEWs) • Kenya (CHPs) • Egypt (Village pioneers-Raedat Refiat (RR)) • Namibia (HEWs) • Uganda (VHTs &CHEWs) • DRC/Zambia/South Africa/Nigeria/Rwanda/Sierra Leone/Mali (CHWs) • Malawi (HSAs) • Burkina Faso (CBHWs) • Mozambique (Agentes Polivalentes Elementares (APEs))	Paper-based tools • Family folder (Ethiopia) • Household registers (Kenya, Egypt, Rwanda, Malawi) • CHWs service Logbook (Kenya) • Simple wall chart templates (Malawi) • Paper registers (Ghana, DRC, Zambia) • Forms/papers (Namibia, South Africa& Ghana) • Surveillance forms (Burkina Faso) Electronic-based tools • Mobile phone applications/technologies/mHealth tools Other sources of data: • Individual health records (health cards and integrated maternal and childcare (MCH) cards) • Assistant chief registers • Community outreach and meetings • Birth and death register • Village register • Under-five register • Household survey/visit form • Community treatment and tracking register • Referral form	• Health Extension program component data (Ethiopia) • Program data related to HIV, TB & Malaria (South Africa, Mozambique & Zambia & Namibia) • Child health data element (DRC, South Africa) • Maternal and child civil registration data (Nigeria & Ghana) • Maternal, Neonatal, and Child Health (MNCH) morbidity & mortality data (Sierra Leone) • Maternal and child health services data (Namibia, Malawi & Uganda) • Demographic, household sanitation, housing, health service utilization and coverage (commonly collected) • Supply chain management data (Kenya & Zambia)	CHWs supervisors • Ethiopia (HEW supervisors/ coordinators) • Kenya (community health assistants/officers (CHAs/CHOs)) • Egypt (RR supervisors) • Namibia (CHW supervisors) • Uganda (Health Centre (HC) III in-charge) • DRC (HC supervisors) • South Africa (Outreach Team Leaders/data captures) • Sierra Leone (CHWs peer supervisors) • Malawi (Senior HSAs) • Burkina Faso (CBHWs supervisors) • Funders department (South Africa)

Note: *CHEWs: Community Health Extension Workers; CHWs: Community Health Workers; CHPs: Community Health Promoters; DRC: Democratic Republic of Congo HEWs: Health Extension Workers; VHTs: Village Health Teams; HSAs: Health Surveillance Assistances; CBHWSs: Community Based Health Workers*

Data collection tools and information collected used by CHWs vary by country and services provided at the community level ( Table 2). CHWs commonly use standardized household registers during house visits to collect community data. Other data collection tools included simple wall charts ^26^, CHW Integrated Daily Activity Register/logbooks ^1^, individual health cards ^1, 27, 28^, and surveillance forms ^29^. The CHWs typically collect household data, including household demographics, sanitation, housing, health service utilization, and coverage ^30– 38^. For instance, in DRC and South Africa ^39^, CBHIS focused on child health data, whereas in Uganda, Namibia, and Malawi, maternal and child health data were captured ^1, 27, 28^. Sierra Leone’s ^40^, Nigeria's ^41^, and Ghana's ^42^ CBHIS includes Maternal, Neonatal, and Child Health (MNCH) mortality and morbidity data to inform health service delivery and development of interventions. Some of the CBHIS in Namibia ^1^, Zambia ^38^, Mozambique ^43^, and South Africa ^39, 44^ collect program-specific data on HIV/AIDS and TB care, malaria data, and households’ eligibility for social support.

***CBHIS data pathways.*** CHWs primarily use paper-based tools for data collection ^38, 42, 45^; however, some countries have adopted electronic-based systems (eCBHIS), such as mobile phone applications and mHealth tools ^41– 53^). In some instances, CHWs must use both manual and eCBHIS methods, as observed in Ethiopia ^47, 54^ and Ghana ^42^. Additionally, mobile technology has been utilized to collect community health data, such as in Kenya, where the mHealth application has been used to collect non-communicable diseases, particularly diabetes and hypertension ^55^, and a simple short message service (SMS) – based reporting to support the supply chain management ^50^. A mobile-based eCBHIS was implemented in Zambia to monitor commodities stock levels ^52^.

In paper-based systems, CHWs record household visits and activities in standardized Federal/National Ministry of Health (MoH) service delivery registers, which are then collated to complete monthly report forms. These report forms their respective catchment areas are then submitted to the supervisors, who aggregate the data in paper-based standardized MoH forms that are in turn submitted to the sub-national office (sub-county, county, district, or regional) for digital entry into the web-based national health information systems, the District Health Information System (DHIS2) ^26, 56– 62^. Notably, digital entry of the paper-based systems into the DHIS2 database happens at the sub-national level ^1, 11, 32^. However, the lack of harmonization of CHW data collection tools and HMIS forms has been identified as a barrier to data capture in HMIS during data submission ^33, 63, 64^.

In electronic-based systems, data on household visits or program-specific indicators are entered electronically by CHWs into electronic forms on the applications installed on their tablets or mobile phones and submitted electronically to the organization’s database or sub-national or national HMIS, DHIS2 ^27, 37, 41, 51, 52^. The electronically aggregated data in the HMIS are made visible and accessible to CHWs supervisors, health managers, and data managers, who review data, trace data errors in data capture, track and analyse data, as well as send electronic feedback notes to CHWs ^41, 51, 52^. Some applications have built-in data validation to ensure the completeness of data ^41, 51^. However, in other instances, CBHIS data are directly conveyed to the department of funders, bypassing health facilities for electronic database recording ^60^.

CBHIS data review/use meetings are intended to also create effective feedback mechanisms across healthcare service levels ^1^. However, the implementation of these mechanisms was often limited to human resource constraints, as observed in Namibia, whereas in DRC, feedback mechanisms were reported to function better in areas with partner support, and in Uganda, feedback was reliant upon the provision of supportive supervision ^1^.

The data flow for the CBHIS and feedback mechanisms varied depending on the country and tools used for data collection, whether paper-based or electronic-based.

***Utilisation of CBHIS data.*** At the national/federal level, the division/department responsible for health information systems receives community health data from sub-national levels, which is then transmitted to the division responsible for community health services within the Ministry of Health (MoH) ^1^. The division of community health services utilizes the data to track the progress of community health programs, create annual health sector performance reports, formulate policies, and provide feedback to decentralized levels. Ideally, all levels of the health system, including community, sub-national, and national, should review and utilise CBHIS data ^1^. However, data producers and users often lack the core competencies of data analysis, interpretation, and synthesis, which, in turn, limit the demand and use of data in decision-making processes ^1, 11, 65^.

CBHIS data is reportedly utilized by various stakeholders, including government entities, NGOs, CBOs, funders, health facilities, community health committees, and healthcare professionals at different levels, to guide decision-making, policy decisions, staffing, commodities supplies, and resource allocation for community health programmes ^26, 29, 52, 57, 64^. In Ethiopia and Malawi, CBHIS data is used to support health extension services ^30, 54, 61, 66, 67^, whereas, in South Africa, CHWs use it for community activities and referrals to service providers ^46^. In Namibia, the MoH uses it to inform future community health programmes ^1^, while health managers in Ethiopia use it to monitor and evaluate community health services ^54, 61^. In Kenya, CBHIS programme data is used to assess interventions ^68^ and design new ones, and in South Africa, regional coordinators use it for programme tracing and planning ^60^. CBHIS data also supports collective activities such as community dialogue in Kenya, South Africa, Malawi, and Ethiopia to address the prevalent challenges in catchment areas/community units ^12, 36, 56, 58^.

Moreover, CBHIS data is utilized in various ways by CHWs and healthcare providers in Ethiopia, Kenya, South Africa, Malawi, and Zambia, such as tracking defaulters for health services and scheduling house visits ^12, 33, 37, 46, 68^, assessing the utilization and coverage of maternal and newborn care services ^69^, institutional delivery of immunization ^59, 69– 71^, monitoring trends in health service delivery and disease prevalence, and implementing mitigation strategies for disease outbreaks ^12, 63^. CHWs also use this data to monitor community health supplies and commodity stock levels ^33, 50, 52^ and plan health resources at the sub-national and national levels ^28, 38, 41^. While CBHIS data is crucial for improving community health programs and outcomes, challenges remain in effectively using data at the community level other than for reporting purposes ^12, 26^.

***Accessibility of CBHIS data and community empowerment.*** Community dialogue is the most widely used strategy for community engagement, empowerment, and access to CBHIS data. Studies conducted in Ethiopia, Kenya, Malawi, and South Africa have reported that community dialogue brings together community members, leaders, representatives, community health committees, CHWs, and health providers to share CBHIS data for priority setting, planning, implementation, evaluation of health interventions/programmes, and consensus in addressing specific community health issues ^36, 46, 56– 58, 68, 69, 72^. CHWs collaborate with community health-level committees to initiate community dialogues. In addition to community dialogue, community members can access CBHIS data through wall charts/chalkboards displayed in community units, health centers, and clinics ^26, 32, 33^. However, a multi-country study across 17 West and Central African countries revealed the lack of CBHIS data accessibility to community members beyond the CHWs, impeding community participation in data utilization ^73^. The included studies have attributed improved health indicators, health service utilization, and health practices, including improved sanitation and hygiene practices, drug adherence, reduced stigma, increased family planning methods, immunization, and maternal delivery to community dialogues ^12, 36, 68^. Nonetheless, evidence directly linking community dialogue to improving specific health indicators, health status, and health practices is limited. Community empowerment in community dialogues tends towards health promotion activities ^36, 56– 58^.

## Discussion

CHS is a crucial aspect of PHC and a vehicle for achieving UHC and other global health SDG priorities. To effectively deliver community health services, a functional and practical CBHIS is essential for countries to track their progress toward PHC and UHC. This scoping review aimed to synthesise evidence on the current practices of CBHIS data generation, data pathways across different health system levels, utilisation of CBHIS data, and accessibility of CBHIS data to communities to empower communities in African countries. The majority of articles reported on CBHIS data generation and use. Most CBHIS utilize paper-based systems, although some countries have adopted electronic/digital systems (eCBHIS) to record and transmit data to sub-national and national HMIS; data pathways vary by country. Multiple stakeholders utilize CBHIS data for decision-making, including policymaking, resource allocation, staffing, programme evaluations, and informing community health programmes and dialogues. Community dialogue is the most common strategy for community engagement, sharing CBHIS data, and empowering communities.

CHWs are crucial in generating data for the CBHIS. Different cadres of CHWs have distinct roles and include data collection, management, and dissemination. Although most countries rely on paper-based systems for data collection, some use electronic-based systems ^1, 37, 45– 47^, or a combination of both ^47, 54^. However, reported challenges included a lack of standardized data collection and compilation tools ^1, 11^, inadequate personnel competencies ^37, 51, 52^, and duplicate data entries in paper-based and electronic forms ^1^, can lead to limited data collection and loss, negatively impacting data quality. As countries transition to digitized systems, it is crucial to provide regular technical and supportive supervision to CHWs to tackle user-related and system-related challenges they face with eCBHIS. Continued training for CHWs on basic Information Communication Technology (ICT) skills, digital tools, and data analysis and use, is still vital to ensure timely, accurate, and complete data entry into eCBHIS ^4, 47, 65, 74, 75^.

The contextual adoption of mobile technology can help with the transition e.g., simple feature phones with simple SMS-based reporting systems have been successfully adopted in Ghana, Kenya, Malawi, and Zambia ^4, 19^. Our review revealed an absence of policy guidance concerning data security and privacy aspects for both paper- and electronic-based CBHIS systems ^11, 41^. For instance, CHWs were obliged to store paper-based data in their homes owing to insufficient storage, leading to lost data forms and the potential breach of confidentiality ^11^. To enhance the security and privacy of CBHIS data in the healthcare sector, countries transitioning to digital systems should develop or update their eCBHIS policy frameworks. These frameworks should address the gaps in data security and privacy, safeguard community data and guide the implementation of data protection principles in eCBHIS ^1, 76^.

CBHIS generates large amounts of data on healthcare services and population health, presenting opportunities for data-driven decision-making in the CHS. While efforts to enhance CBHIS have primarily focused on digitalization and improving data collection and quality, particularly at the community level, there is a disproportionate emphasis on the technical aspects of enabling data use ^74, 77^. The ultimate goal of CBHIS is to translate data into action, address health challenges, and improve the access and quality of community health services ^77^. We indicate that CBHIS data can be utilized in health system and service outcomes, health resource allocation, and administrative decisions ^45, 54, 57, 59, 66, 78^; however, there was limited evidence on the impact of the data-driven decision-making approach in the included studies. Various challenges impede the utilisation of CBHIS data, such as fragmented reporting systems ^13^, poor coordination between data producers and users ^12, 13^, variations in the decentralization of community health decisions ^14^, and limited capacity of data producers and users and utilize the data for decision-making ^1, 11, 65^.

To ensure sustainable demand and use of data in decision-making, it is essential to develop the capacity of data producers and users in core competencies, such as data analysis, interpretation, and synthesis, at all levels of the health system, including the CHS. Investing in capacity-building for data producers and users on critical competencies can facilitate the functioning of CBHIS ^79^. Lippeveld (2017) identified many barriers to data use related to organizational and behavioural factors ^77^. The information use culture can act as both a barrier ^12, 26^ and a facilitator ^80^ in data utilization. Negative organizational behaviour, such as the pressure senior health managers exert on providers to meet unrealistic service delivery targets, has contributed to false reporting and the denial of existing service delivery problems. Conversely, community-led monitoring of health service delivery data has been demonstrated to promote positive organization behaviour by enhancing the culture of information ^77, 80^.

Community participation in health information generation and dissemination has been shown to increase community engagement and health information sharing and foster health system responsiveness through community activism ^20, 80– 82^. However, community members face barriers to accessing and using health information. A multi-country study across 17 West and Central African nations found that community members lacked access to CBHIS data beyond that of CHWs, which hindered their participation in data utilisation ^73^. This limits the involvement of end-users of care in developing interventions that align with local needs and are informed by local knowledge and priorities in a more effective and transformative way that helps empower marginalized and vulnerable population groups. Community data dissemination has shown positive results in various initiatives ^80, 83, 84^. For instance, a randomised field experiment in nine districts in Uganda revealed that granting communities access to data increased their involvement, accountability, and community-led monitoring of PHC services ^80^. Consequently, service utilisation and health outcomes improved significantly. This intervention emphasises the magnitude of community participation and a bottom-up approach to enhancing CHS service delivery and health outcomes. Integrating this approach with a structured top-down approach can lead to even better results ^80^.

The results of our review carry with them some implications. The CHS require the availability of good-quality data, however, this on its own is insufficient to support the use of data in the CHS and broader health systems management decision-making. Although studies included in our review reported the utilization of CBHIS data, there are deficiencies in comprehending the extent to which it's used or integrated in decision-making processes and policy formulation. The health authorities and practitioners may need to consider implementing interventions that explicitly focus on improving the link between CBHIS data collection and the use of data for decision-making. CHS activities, policies and guidelines may need to focus on capacity building of data producers and users in data management and data use competencies, including analysis, synthesis, interpretation, critical review of data, and data-informed decision making ^1, 11, 65^. In addition, there is a need to focus on organizational culture and practice of monitoring, evaluation, and communication of data use interventions, and that encourages health managers, frontline health providers and users of health services, to take responsibility for using data to inform decision making ^7, 77, 80^.

Our review suggests that there is limited access to CBHIS data beyond community dialogues and wall charts in community health units. Accessibility of CBHIS data to the community is essential to foster community participation in community health activities and accountability. An experimental study on information intervention in Uganda shows that disseminating data to community members can enhance community participation in CHS services, empower them and promote accountability of health providers at the community level ^80^. However, there is a gap in studying the impact of community participation and empowerment on health outcomes.

There is a large and diverse body of literature on CBHIS data generation/production (data sources, data management, information products and dissemination) and systems performance (data quality and data use). However, there is a research gap on the links between data collection and data use, and between data use and systems impact, as well as components needed for the design and evaluation of CBHIS, to effectively support health system management decision-making. Implementation research approaches may also help understand data-driven decision-making mechanisms in operational settings ^7^.

### Strengths and limitations

We conducted a systematic and thorough evaluation of the existing literature. Our approach involved conducting a comprehensive literature search, employing duplicate article screening, and selecting articles by independent reviewers, with senior reviewers verifying and ensuring quality control. However, our review had some limitations. First, we only included published articles and grey literature, which may have led to the exclusion of other relevant documentation on CBHIS. Second, we did not consider non-English studies or grey literature, which could have resulted in the exclusion of articles from non-English-speaking African countries that may have been relevant to our review.

## Conclusion

While there is a focus on enhancing data collection, processing, quality, and digitization of CBHIS, there is a need to shift the emphasis towards enabling data utilisation at the community level and community empowerment. Community involvement and empowerment are mainly achieved through community dialogue, but it should move beyond supply-side-driven health promotion activities and enhance demand-driven interventions to foster community accountability and tailor interventions to community needs. Demand-driven initiatives can promote community participation in CBHIS, community empowerment, and health activism. The renewed commitment to PHC presents an opportunity to optimise the functionality of CBHIS and accelerate progress towards UHC and other health-related SDGs.

## Data Availability

All data underlying the results are available as part of the article and no additional source data are required. Havard Dataverse: Replication Data for: Examining the Development and Utilisation of Community-Based Health Information Systems (CBHIS) in Africa: A Scoping Review https://doi.org/10.7910/DVN/ZH5JK8 ^25^ This project includes the following extended data: Additional File 1 (information search strategy) Additional File 2 (data extraction form) Additional File 3 (characteristics of included studies). Havard Dataverse: PRISMA_ScR Checklist for ‘Examining the development and utilisation of Community-Based Health Information Systems (CBHIS) in Africa: A Scoping Review’. https://doi.org/10.7910/DVN/ZH5JK8 ^25^. Data are available under the terms of the Creative Commons Attribution 4.0 International license (CC-BY 4.0).
