# Supplementary material for: Community-Based Health Information Systems in Africa: A Scoping Review of Data Generation, Utilization, and Community Empowerment
Source: Wellcome Open Res. 2024 Oct 21;9:485. Originally published 2024 Aug 15. [Version 3] doi: 10.12688/wellcomeopenres.22780.3 (PMC11403289; doi:10.12688/wellcomeopenres.22780.3)
Supplement: Peer review history

# Reviewer report: Reviewer response for version 3

**DOI:** 10.21956/wellcomeopenres.25617.r106814

**Published:** 2024-11-01

**Authors:** Robinson Karuga

**Affiliations:** 1 LVCT Health, Nairobi, Kenya

The updated version reads well and is therefore approved.

Are the rationale for, and objectives of, the Systematic Review clearly stated?

Yes

Is the statistical analysis and its interpretation appropriate?

Not applicable

If this is a Living Systematic Review, is the ‘living’ method appropriate and is the search schedule clearly defined and justified? (‘Living Systematic Review’ or a variation of this term should be included in the title.)

Not applicable

Are sufficient details of the methods and analysis provided to allow replication by others?

Yes

Are the conclusions drawn adequately supported by the results presented in the review?

Yes

Reviewer Expertise:

Health Policy and Systems Research

I confirm that I have read this submission and believe that I have an appropriate level of expertise to confirm that it is of an acceptable scientific standard.

# Reviewer report: Reviewer response for version 3

**DOI:** 10.21956/wellcomeopenres.25617.r106815

**Published:** 2024-10-24

**Authors:** D'Ambruoso Lucia

**Affiliations:** 1 Medical Sciences and Nutrition, University of Aberdeen, Aberdeen, UK

Thank you for the detailed response to the review. The substantive points have been addressed in Version 3. I have no further comments.

Are the rationale for, and objectives of, the Systematic Review clearly stated?

Yes

Is the statistical analysis and its interpretation appropriate?

Not applicable

If this is a Living Systematic Review, is the ‘living’ method appropriate and is the search schedule clearly defined and justified? (‘Living Systematic Review’ or a variation of this term should be included in the title.)

Not applicable

Are sufficient details of the methods and analysis provided to allow replication by others?

Yes

Are the conclusions drawn adequately supported by the results presented in the review?

Partly

Reviewer Expertise:

Public health, health policy and systems research, community-led health

I confirm that I have read this submission and believe that I have an appropriate level of expertise to confirm that it is of an acceptable scientific standard.

# Reviewer report: Reviewer response for version 2

**DOI:** 10.21956/wellcomeopenres.25479.r100268

**Published:** 2024-09-25

**Authors:** Dan Kaseje

**Affiliations:** 1 Tropical Institute of Community Health and Development, Great Lakes University of Kisumu, Kisumu, Kenya

I have read this submission. I believe that I have an appropriate level of expertise to confirm that it is of an acceptable scientific standard. It is approved **.**

Are the rationale for, and objectives of, the Systematic Review clearly stated?

Yes

Is the statistical analysis and its interpretation appropriate?

Yes

If this is a Living Systematic Review, is the ‘living’ method appropriate and is the search schedule clearly defined and justified? (‘Living Systematic Review’ or a variation of this term should be included in the title.)

Yes

Are sufficient details of the methods and analysis provided to allow replication by others?

Yes

Are the conclusions drawn adequately supported by the results presented in the review?

Partly

Reviewer Expertise:

NA

I confirm that I have read this submission and believe that I have an appropriate level of expertise to confirm that it is of an acceptable scientific standard.

# Reviewer report: Reviewer response for version 1

**DOI:** 10.21956/wellcomeopenres.25084.r95037

**Published:** 2024-09-20

**Authors:** Dan Kaseje

**Affiliations:** 1 Tropical Institute of Community Health and Development, Great Lakes University of Kisumu, Kisumu, Kenya

The work is insightful for publication and contributing to the body of knowledge in the area of CBHIS.

**Title:**  The title of the review should be precise and consistent with the problem statement and objectives

 The title reads: Examining the development and utilization of Community-Based Health Information Systems (CBHIS) in Africa: A Scoping Review.  The problem statement and objectives focus on generation and utilization of CBHIS information, leaving out the development of CBHIS, reflected on the title. Authors to consider excluding “development from the title” and thus harmonize the title with problem statement, the objectives, results and discussion.

**Scope: **The title states reflects the scope to be the review to be Africa, yet the work reviewed as reflected by results , appear to be Sub Saharan Africa, no single study from North Africa, reflected.

**Abstract:**

 The objective reads “This scoping review aims to comprehensively examine the use of CBHIS in African countries, focusing on data generation, pathways, utilization of CBHIS data, community accessibility to the data and use” The content does not reflect CBHIS pathways. There is need to highlight pathways, or exclude it from the objectives.

**Background: **The problem statement is clear, stating why this study was necessary and what it aimed to contribute to the field. The section could end with a very brief statement of what is being reported in the article.

**Methods:  **The method section has been well written giving sufficient information and elaborating the design of the study including theoretical frameworks applied in data collection analysis.

 The inclusion of grey literature is commendable although some reflection on the strength of evidence, given the numerous sources of error involved as compared to scientifically designed works.

**Results: **Going through the results section, which is well written but the cannot miss to notice the relative over-representation of articles from Kenya, East Africa, and could wonder about some  bias in literature access. A statement to demonstrating that articles from the other regions of Africa had equal chance of being included, may be useful.

 To help the reader have a glimpse of the achievements and challenges, study type, country where the study was undertaken will be good at the end of the section to have a table or a matrix summarizing the findings based themes or review objectives.

**Discussion: **Could be structured way, as follows: a) statement of principal findings by objectives; b) strengths and weaknesses of the review; c) contextualizing findings in context of similar reviews hence d) contribution to new knowledge

**Conclusion:** Is well written but could provide a brief summarize of the key findings, potential implications and the way forward.

Are the rationale for, and objectives of, the Systematic Review clearly stated?

Yes

Is the statistical analysis and its interpretation appropriate?

Yes

If this is a Living Systematic Review, is the ‘living’ method appropriate and is the search schedule clearly defined and justified? (‘Living Systematic Review’ or a variation of this term should be included in the title.)

Yes

Are sufficient details of the methods and analysis provided to allow replication by others?

Yes

Are the conclusions drawn adequately supported by the results presented in the review?

Partly

Reviewer Expertise:

Health systems research and development including Community Based Health Care

I confirm that I have read this submission and believe that I have an appropriate level of expertise to confirm that it is of an acceptable scientific standard, however I have significant reservations, as outlined above.

# Response

**Published:** 2024-10-08

**Authors:** Moriasi Nyanchoka

**Affiliations:** Health Economics Research Unit, KEMRI-Wellcome Trust Research Programme Nairobi, Nairobi, Nairobi County, Kenya

Thank you for reviewing our article. Please find our responses to your comments below. **TITLE** **Comment:** The title of the review should be precise and consistent with the problem statement and objectives. The title reads: Examining the development and utilization of Community-Based Health Information Systems (CBHIS) in Africa: A Scoping Review.  The problem statement and objectives focus on generation and utilization of CBHIS information, leaving out the development of CBHIS, reflected on the title. Authors to consider excluding “development from the title” and thus harmonize the title with problem statement, the objectives, results and discussion.

**Response: **Thank you for highlighting this. We have revised this. The title now reads “Community-Based Health Information Systems in Africa: A Scoping Review of Data Generation, Utilization, and Community Empowerment”

**SCOPE Comment: **The title states reflects the scope to be the review to be Africa, yet the work reviewed as reflected by results , appear to be Sub Saharan Africa, no single study from North Africa, reflected.

**Response:  **Our search strategy aimed to capture studies across the entire African continent. However, we only obtained data from Egypt. This limited representation could be attributed to our inclusion criteria restricting articles to those published in English, potentially excluding research from many non-English speaking North African countries. However, we acknowledge this in the limitation section. **“**Second, we did not consider non-English studies or grey literature, which could have resulted in the exclusion of articles from non-English-speaking African countries that may have been relevant to our review”.

**ABSTRACT** **Comment: **The objective reads “This scoping review aims to comprehensively examine the use of CBHIS in African countries, focusing on data generation, pathways, utilization of CBHIS data, community accessibility to the data and use” The content does not reflect CBHIS pathways. There is need to highlight pathways, or exclude it from the objectives.

**Response: **We have incorporated additional content regarding CBHIS pathways in the "Synthesis of Results" subsection of the Results section. Figure 1 provides a visual representation of data flow.

**BACKGROUND** **Comment: **The problem statement is clear, stating why this study was necessary and what it aimed to contribute to the field. The section could end with a very brief statement of what is being reported in the article

**Response: **Thank you for highlighting this. We have added a brief statement on this. The section now reads “We aim to address the gap in these aspects and inform efforts to enhance the CHS, ultimately contributing to improved community health service coverage and tracking progress towards UHC and other health-related SDGs. To comprehensively understand CBHIS functionality and its potential impact on community health outcomes, we systematically examined four key aspects: data generation processes, data flow pathways, CBHIS data utilisation, and community access and utilisation of this data for empowerment.”

**RESULTS** **Comment: **Going through the results section, which is well written but the cannot miss to notice the relative over-representation of articles from Kenya, East Africa, and could wonder about some  bias in literature access. A statement to demonstrating that articles from the other regions of Africa had equal chance of being included, may be useful.

**Response: **Thank you for highlighting this. We have incorporated a statement on this. The statement now reads “We synthesized 55 studies from 27 African countries, primarily Eastern and Southern Africa, followed by the Wester African region. Although the literature review considered publications from all African countries, Northern Africa was represented by only a single article from Egypt”.

**DISCUSSION** **Comment: **Could be structured way, as follows: a) statement of principal findings by objectives; b) strengths and weaknesses of the review; c) contextualizing findings in context of similar reviews hence d) contribution to new knowledge

**Response: **The structure of our discussion follows the PRISMA Scoping Review and reporting guidelines. It begins with a synthesis of the findings for each focal area: data generation, data pathways, data utilisation, and community accessibility and empowerment. The second to last paragraph addresses the limitations of the review, as per the PRISMA Scoping Review Guidelines.                     

**CONCLUSION** **Comment: ** Is well written but could provide a brief summarize of the key findings, potential implications and the way forward

**Response: **We have revised this section to provide a summary of key findings and take-aways. We have discussed the implications of our findings in the discussion section, in the third last paragraph. The section now reads “CBHIS are transitioning from paper-based to electronic-based systems, aiming to improve data collection and pathway processes. However, several challenges, including fragmented reporting and limited capacity, impede the effective utilisation of CBHIS data. The successful adoption of CBHIS requires considering resource allocation and technology adoption within the context of the CHS and the broader health system. While efforts focus on the digitization of CBHIS and enhancing data generation, overlooking other factors may hinder effectiveness. Developing core competencies in data analysis and interpretation among data producers and users across all health system levels is crucial.  ,  Community dialogue, while valuable for engagement, requires a shift from provider-driven/supply-side health promotion activities to community-driven/demand-side interventions. Community-driven approaches can enhance community participation in CBHIS, accountability, empowerment, health activism, and tailoring interventions to local needs. The renewed commitment to PHC presents an opportunity to optimise the functionality of CBHIS and accelerate progress towards UHC and other health-related SDGs.”

# Reviewer report: Reviewer response for version 1

**DOI:** 10.21956/wellcomeopenres.25084.r95042

**Published:** 2024-09-18

**Authors:** D'Ambruoso Lucia

**Affiliations:** 1 Medical Sciences and Nutrition, University of Aberdeen, Aberdeen, UK

Review

**Examining the development and utilisation of Community-Based Health Information Systems (CBHIS) in Africa: A Scoping Review**

**OVERALL COMMENTS**

 This paper presents a literature review on community-based health information systems in low- and middle-income countries. The analysis provides a granular account of the generative, processing, utilisation and accessibility pathways across a range of African nation states, appraises these findings, and derives recommendations. The paper is important, original and worthy of indexing. The following are some relatively minor comments, which I hope are of use to the authors to further strengthen the piece. N.B.: I am not a professional systematic reviewer and cannot comment on the methodology in anything other than general terms.

 Overall, and considering the opening arguments raise the important issue of data infrastructure fragmentation, including through the mixed influence of development partners and donor institutions, it would be useful to understand the extent to which data generated by CBHISs are used (primarily at least) locally and/or domestically and/or regionally/internationally. If this is not reported, then that is also a finding!

 Secondly, the political nature of data, may be worth considering - issues such as and including how to progress beyond ‘naïve rationality’ towards fuller views of community data and data use. This is raised explicitly in the paper but deserves more attention. What are the risks, as well as benefits for communities around CBHIS?

 Thirdly, there are some powerful statements about data use (and the political nature of data) however it is not clear whether these are findings from the empirical work, this should be clarified (see specific comments, below).

 Fourth, I was surprised Rwanda does not feature in the findings, considering the expansion of the CHW programme to NCDs.

 Finally, the discussion section could be strengthened by ‘unpacking’ some key concepts (participation, empowerment, accountability etc) and arguments further, please see specific comments. On page 9 for example, the statement ‘data pathways vary by country’ could be usefully supplemented with additional detail. I would also encourage the authors to balance the ‘inward’ and ‘outward gaze’ in the recommendations. The systems and implementation challenges are not just local problems. It follows that it is unlikely that the challenges identified can resolved entirely by front line service providers, particularly CHWs. The Discussion would be usefully balanced with critical reflections on, and recommendations for, national, regional, and international levels.

**SPECIFIC COMMENTS**

**Methods**

1. Throughout, minor comment – data is a plural term.
2. A map showing where CBHIS are reported on might be a useful visual representation of the evidence base to consider including.
3. Page 5 – data items and charting: the data extraction themes are great suggest numbering them to help readers navigate the content.
4. Page 5: the data extraction theme ‘sources of CBHIS data’ stated in the methods seems to correspond with ‘Characteristics of sources of evidence’, although it may correspond with ‘general study characteristics’.  All other extraction themes are clearly presented and aligned to the methods. The ‘sources of CBHIS data’ could benefit from being more clearly reported.

**Results**

1. Page 5, ‘CBHIS data generation’ and Table 2: it would be useful to understand whether and how development partner programmes and/or statutory services feature in types of community-based health information. Considering, who are these data (primarily) for.
2. Page 8: as per the comment, above, the statement ‘CBHIS data is reportedly utilized by various stakeholders, including government entities, NGOs, CBOs, funders, health facilities, community health committees, and healthcare professionals at different levels, to guide decision-making, policy decisions, staffing, commodities supplies, and resource allocation for community health programmes’ would benefit from more fine-grained detail.
3. Page 8: while the term ‘defaulters’ is in popular use especially at front line service delivery level, international consensus is that it is stigmatising and ‘people lost to follow up’ is the preferred term. Suggest the authors acknowledge this in some way. Supporting sources include: Stop TB Language Matters’ https://www.stoptb.org/sites/default/files/languageguide_forweb20131110.pdf , https://conf2022.theunion.org/wp-content/uploads/2022/05/stbp_words-matter_screen-ready.pdf
4. Page 9, the theme ‘empowerment’ would benefit from a little development (space allowing). A notion of what is meant by empowerment together with some grounded consideration of the functionality of existing community dialogue/governance spaces. For instance, in South Africa, while clinic and health committees are well established local governance processes, there is evidence that they have limited functionality. See Hove et al. https://doi.org/10.1080/16549716.2021.2004730 ^(1)^, Haricharan et al. https://doi.org/10.1017/s1463423621000323 ^(2)^ and https://doi.org/10.1017/s146342362100027x ^(3)^
5. Page 9, similarly the statement ‘limited. Community empowerment in community dialogues tends towards health promotion activities’ needs to be rationalised/evidenced/substantiated a little further.

**Discussion**

1. Page 9: the statement ‘data pathways vary by country’ could be usefully supplemented with additional detail – the analysis provides a granular account of how the generative, processing, utilisation and accessibility pathways vary by several other factors. Similarly, the statement about multiple stakeholders use CBHIS data could provide a little more nuance. These statements, while useful to summarise the findings are very general.
2. Page 9: the statement on ‘continued training for CHWs’ is useful but also would benefit from more detail. Who should conduct this training? Statutory services? What are the opportunity costs of this? What is the role of development partners, here? The recommendations are good and grounded in the findings but could be usefully unpacked a little further.
3. Page 9: the finding on storage of health information in CHWs’ residences is powerful, and should be presented in the Results section, and then followed up in the Discussion with a thorough critique. In South Africa, for example, the finding could be considered in terms of the relatively new POPIA legislation on data protection, and in terms of the struggle of CHWs; who are recognised/centralised in PHC policy and strategy but poorly supported and resourced at operational and implementation levels.
4. Page 9: the discussion point on the ‘disproportionate emphasis on technical aspects of enabling data use’ is great, and again could be unpacked a little further. There is evidence, again in South Africa, of district health information systems being costly, producing poor quality information with limited uptake and with ‘upwards’ data flows, missing opportunities to realise the potential of data at local level. Some supporting references on DHIS: Hotchkiss et al. https://doi.org/10.1186/1472-6963-10-188 ^(4)^; Rouamba et al. https://doi.org/10.1038/s41598-020-73601-3 ^(5)^; Nshimyiryo et al. https://doi.org/10.1371/journal.pone.0235823 ^(6)^
5. More broadly on this point, some attention to the relationships documented in the literature on the connection of CBHIS to DHIS and HMIS would be useful.
6. Page 9: the statement ‘We indicate that CBHIS data can be utilized in health system and service outcomes, health resource allocation, and administrative decisions’ is not quite clear. Does this mean that, in theory, CBHIS can support data-informed decision making, but in practice there are many implementation challenges? The description of these challenges at the end of the passage is great and could usefully be supplemented with the upward accountability (and data) flows in many health systems, together with the fragmentation that can related at least in part to vertical donor and development partner programming.
7. Page 9: the core recommendations seem to focus ‘internally’ on improving the capabilities of CHWs for collection, analysis and reporting. This is in part founded and reasonable however, the recommendation could be strengthened by balancing it with an ‘outward gaze’, considering the national and subnational health systems challenges, as well as the supranational drivers as noted through these comments. How feasible are these recommendations, considering the implementation realities of PHC?
8. Page 9: the statement ‘Negative organizational behaviour, such as the pressure senior health managers exert on providers to meet unrealistic service delivery targets, has contributed to false reporting and the denial of existing service delivery problems’ is another powerful statement. However, it is unclear if this is a finding from the data, or from elsewhere. Please reference and relate to a supporting source, and if this is the data set, then present as a finding. Relates to the political nature of data, and may be worth considering issues such as and including how to progress beyond ‘naïve rationality’ towards a more rounded view of community data and data use, including an appreciation of the political nature of local, routine health data.
9. Page 10: the discussion on community participation, as above, may usefully refer to evidence on the existence of spaces and processes, but their limited functionality in practice, and consider the implications of this.

**Conclusion**

1. Page 10: the concluding statement needs some unpacking and grounding I, which could be done in the Discussion section - ‘Community involvement and empowerment are mainly achieved through community dialogue, but it should move beyond supply-side-driven health promotion activities and enhance demand-driven interventions to foster community accountability and tailor interventions to community needs’. As above, it would be useful to outline what is meant by community participation, empowerment and accountability. What community dialogue is and means, how it is done and the challenges it faces in practice and in reality.

End of review

Are the rationale for, and objectives of, the Systematic Review clearly stated?

Yes

Is the statistical analysis and its interpretation appropriate?

Not applicable

If this is a Living Systematic Review, is the ‘living’ method appropriate and is the search schedule clearly defined and justified? (‘Living Systematic Review’ or a variation of this term should be included in the title.)

Not applicable

Are sufficient details of the methods and analysis provided to allow replication by others?

Yes

Are the conclusions drawn adequately supported by the results presented in the review?

Partly

Reviewer Expertise:

Health policy and systems research; participatory and qualitative methodologies; social policy; health inequalities

I confirm that I have read this submission and believe that I have an appropriate level of expertise to confirm that it is of an acceptable scientific standard, however I have significant reservations, as outlined above.

## References

[1] : Lessons from community participation in primary health care and water resource governance in South Africa: a narrative review. *Glob Health Action* .2022;15(1) : 10.1080/16549716.2021.2004730 2004730 10.1080/16549716.2021.2004730 34994680 PMC8745361

[2] : Effective and meaningful participation or limited participation? A study of South African health committee legislation. *Prim Health Care Res Dev* .2021;22: 10.1017/S1463423621000323 e28 10.1017/S1463423621000323 34109933 PMC8220345

[3] : The role of community participation in primary health care: practices of South African health committees. *Prim Health Care Res Dev* .2021;22: 10.1017/S146342362100027X e31 10.1017/S146342362100027X 34127167 PMC8220489

[4] : Evaluation of the Performance of Routine Information System Management (PRISM) framework: evidence from Uganda. *BMC Health Serv Res* .2010;10: 10.1186/1472-6963-10-188 188 10.1186/1472-6963-10-188 20598151 PMC2904760

[5] : Addressing challenges in routine health data reporting in Burkina Faso through Bayesian spatiotemporal prediction of weekly clinical malaria incidence. *Sci Rep* .2020;10(1) : 10.1038/s41598-020-73601-3 16568 10.1038/s41598-020-73601-3 33024162 PMC7538437

[6] Nshimyiryo A, Health management information system (HMIS) data verification: A case study in four districts in Rwanda. 10.1371/journal.pone.0235823 PMC736746832678851

# Response

**Published:** 2024-10-08

**Authors:** Moriasi Nyanchoka

**Affiliations:** Health Economics Research Unit, KEMRI-Wellcome Trust Research Programme Nairobi, Nairobi, Nairobi County, Kenya

Thank you for your review of our manuscript. Please find our responses to your comments below.

**METHODS** **Comment: **Throughout, minor comment – data is a plural term

**Response: **We have corrected this throughout the methods section.

**Comment: **A map showing where CBHIS are reported on might be a useful visual representation of the evidence base to consider including

**Response: **We have included Figure 1 to provide a visual representation of data flow.

**Comment: **Page 5 – data items and charting: the data extraction themes are great suggest numbering them to help readers navigate the content

**Response: **We have incorporated numbering to guide readers. The section now reads “We extracted data on the following aspects: 1) general study characteristics; 2) sources of CBHIS data; 3) data generation; 4) pathways through which data were processed; 5) utilisation of CBHIS data; and 6) community accessibility to CBHIS data and empowerment.”

**Comment**: Page 5: the data extraction theme ‘sources of CBHIS data’ stated in the methods seems to correspond with ‘Characteristics of sources of evidence’, although it may correspond with ‘general study characteristics’.  All other extraction themes are clearly presented and aligned to the methods. The ‘sources of CBHIS data’ could benefit from being more clearly reported.

**Response: **We have added a theme on this (a sub-heading) under the synthesis of results (page 8) to correspond to themes highlighted in the methods section. The section now reads “ ***CBHIS data sources.*** Data collection tools and information collected used by CHWs vary by country and services provided at the community level (Table 2). CHWs commonly use standardized household registers during house visits to collect community data. Other data collection tools included simple wall charts, CHW Integrated Daily Activity Register/logbooks, individual health cards, and surveillance forms. The CHWs typically collect household data, including household demographics, sanitation, housing, health service utilization, and coverage”.

**Comment: **Page 5, ‘CBHIS data generation’ and Table 2: it would be useful to understand whether and how development partner programmes and/or statutory services feature in types of community-based health information. Considering, who are these data (primarily) for. **Response: **We acknowledge that this is crucial **. **This crucial element is addressed in the results section under the heading “CBHIS data pathways” and  "Utilization of CBHIS."

**Comment: **Page 8: as per the comment, above, the statement ‘CBHIS data is reportedly utilized by various stakeholders, including government entities, NGOs, CBOs, funders, health facilities, community health committees, and healthcare professionals at different levels, to guide decision-making, policy decisions, staffing, commodities supplies, and resource allocation for community health programmes’ would benefit from more fine-grained detail. **Response: **We have revised this statement. The statement now reads “The CBHIS data serve multiple stakeholders in healthcare. Government entities use it for policy decisions, resource allocation, and workforce planning. NGOs and CBOs leverage these data to design targeted interventions, whereas funders allocate resources based on these data. Health facilities rely on CBHIS data to inform service delivery strategies and optimise supply management.  Community health committees leverage this data for advocacy and community engagement. Healthcare professionals incorporate CBHIS data into their decision-making processes to enhance service delivery. This diverse utilisation highlights the importance of improving community health outcomes across sectors.”

**Comment: **Page 8: while the term ‘defaulters’ is in popular use especially at front line service delivery level, international consensus is that it is stigmatising and ‘people lost to follow up’ is the preferred term. Suggest the authors acknowledge this in some way. Supporting sources include: Stop TB Language Matters’

**Response: **Thank you for highlighting this point. We have corrected this. The statement now reads “ Moreover, CBHIS data is utilized in various ways by CHWs and healthcare providers in Ethiopia, Kenya, South Africa, Malawi, and Zambia, such as tracking people lost to follow-up for health services and scheduling house visits”

**Comment: **Page 9, the theme ‘empowerment’ would benefit from a little development (space allowing). A notion of what is meant by empowerment together with some grounded consideration of the functionality of existing community dialogue/governance spaces. For instance, in South Africa, while clinic and health committees are well established local governance processes, there is evidence that they have limited functionality.

**Response: **We have added a description of community empowerment to improve clarity in these subsections.  The statement now reads “Community empowerment extends beyond the involvement, participation, or engagement of communities. It encompasses enhancing individual self-care and lifestyle choices; addressing sociopolitical power dynamics; supporting community-driven priorities, and implementing strategies to improve health and reduce inequities”

**Comment: **Page 9, similarly the statement ‘limited. Community empowerment in community dialogues tends towards health promotion activities’ needs to be rationalised/evidenced/substantiated a little further.

**Response: **We have revised this. The statement now reads “Community empowerment in these community dialogues tends towards provider-led health promotion activities rather than community-driven/demand-driven interventions that foster community accountability and tailor interventions to community needs.”  

**DISCUSSION** **Comment: **Page 9: the statement ‘data pathways vary by country’ could be usefully supplemented with additional detail – the analysis provides a granular account of how the generative, processing, utilisation and accessibility pathways vary by several other factors. Similarly, the statement about multiple stakeholders use CBHIS data could provide a little more nuance. These statements, while useful to summarise the findings are very general. **Response: **We have incorporated Figure 1. Figure 1 illustrates the data flow and is referenced in the "CBHIS data pathways" subsection of the results

**Comment: **Page 9: the statement on ‘continued training for CHWs’ is useful but also would benefit from more detail. Who should conduct this training? Statutory services? What are the opportunity costs of this? What is the role of development partners, here? The recommendations are good and grounded in the findings but could be usefully unpacked a little further.

**Response: **We have revised this statement. The statement now reads “To address these challenges, ongoing training of CHWs by Ministries of Health and partners is crucial. This training should be comprehensive and cover various aspects, including basic Information Communication Technology (ICT) skills, digital tools usage, and data analysis. Moreover, targeted training is crucial for timely, accurate, and complete data entry into eCBHIS.”

**Comment: **Page 9: the finding on storage of health information in CHWs’ residences is powerful, and should be presented in the Results section, and then followed up in the Discussion with a thorough critique. In South Africa, for example, the finding could be considered in terms of the relatively new POPIA legislation on data protection, and in terms of the struggle of CHWs; who are recognised/centralised in PHC policy and strategy but poorly supported and resourced at operational and implementation levels.

**Response:  **We have added this in the results section, under “data pathways”. The added statement reads “ The utilisation of paper-based tools introduces additional obstacles, including stockouts of these tools and insufficient storage capacities. CHWs frequently resort to storing data in their residences. This practice not only increases the risk of data loss but also compromises the confidentiality of sensitive information”.

**Comment: **Page 9: the discussion point on the ‘disproportionate emphasis on technical aspects of enabling data use’ is great, and again could be unpacked a little further. There is evidence, again in South Africa, of district health information systems being costly, producing poor quality information with limited uptake and with ‘upwards’ data flows, missing opportunities to realise the potential of data at local level. Some supporting references on DHIS: Hotchkiss et al.

**Response: **We have revised this statement. We further expound on this in the next paragraph. This statement now reads “While efforts to enhance CBHIS have primarily focused on digitization and improving data collection and quality, particularly at the community level, there is a disproportionate emphasis on the technical aspects of enabling data use, overlooking other factors may hinder its effectiveness. Failure to consider critical elements, such as data analysis and interpretation capabilities across various levels of the health system, may impede the overall effectiveness”.

**Comment: **Page 9: the statement ‘We indicate that CBHIS data can be utilized in health system and service outcomes, health resource allocation, and administrative decisions’ is not quite clear. Does this mean that, in theory, CBHIS can support data-informed decision making, but in practice there are many implementation challenges? The description of these challenges at the end of the passage is great and could usefully be supplemented with the upward accountability (and data) flows in many health systems, together with the fragmentation that can related at least in part to vertical donor and development partner programming.

**Response: **We have revised this statement. The statement now reads “We indicate that CBHIS data can inform health system outcomes and resource allocation and support administrative decision-making processes. Although CBHIS data offer valuable insights, empirical evidence demonstrating its impact on data-driven decision-making remains limited. In practice, several challenges impede the effective utilization of CBHIS data, including fragmented reporting systems, inadequate coordination between data producers and users, varied decentralization of community health decision-making, and limited capacity to use data effectively”

**Comment: **Page 9: the statement ‘Negative organizational behaviour, such as the pressure senior health managers exert on providers to meet unrealistic service delivery targets, has contributed to false reporting and the denial of existing service delivery problems’ is another powerful statement. However, it is unclear if this is a finding from the data, or from elsewhere. Please reference and relate to a supporting source, and if this is the data set, then present as a finding. Relates to the political nature of data, and may be worth considering issues such as and including how to progress beyond ‘naïve rationality’ towards a more rounded view of community data and data use, including an appreciation of the political nature of local, routine health data.

**Response: **We have cited a source to support this statement and the following claim. While not directly stemming from our review findings, the referenced article highlights additional complexities in institutional information-use culture. The statement now reads “Negative organizational behaviour, such as the pressure senior health managers exert on providers to meet unrealistic service delivery targets, has contributed to false reporting and the denial of existing service delivery problems.”

**CONCLUSION** **Comment: **Page 10: the concluding statement needs some unpacking and grounding I, which could be done in the Discussion section - ‘Community involvement and empowerment are mainly achieved through community dialogue, but it should move beyond supply-side-driven health promotion activities and enhance demand-driven interventions to foster community accountability and tailor interventions to community needs’. As above, it would be useful to outline what is meant by community participation, empowerment and accountability. What community dialogue is and means, how it is done and the challenges it faces in practice and in reality.

**Response: **We have further revised this section. A new section has been added to the results, under the theme 'Accessibility of CBHIS data and community empowerment', to clarify the concept of community empowerment in the context of this study. The section now reads “CBHIS are transitioning from paper-based to electronic-based systems, aiming to improve data collection and pathway processes. However, several challenges, including fragmented reporting and limited capacity, impede the effective utilisation of CBHIS data. The successful adoption of CBHIS requires considering resource allocation and technology adoption within the context of the CHS and the broader health system. While efforts focus on the digitization of CBHIS and enhancing data generation, overlooking other factors may hinder effectiveness. Developing core competencies in data analysis and interpretation among data producers and users across all health system levels is crucial.  ,  Community dialogue, while valuable for engagement, requires a shift from provider-driven/supply-side health promotion activities to community-driven/demand-side interventions. Community-driven approaches can enhance community participation in CBHIS, accountability, empowerment, health activism, and tailoring interventions to local needs. The renewed commitment to PHC presents an opportunity to optimise the functionality of CBHIS and accelerate progress towards UHC and other health-related SDGs.”

# Reviewer report: Reviewer response for version 1

**DOI:** 10.21956/wellcomeopenres.25084.r95036

**Published:** 2024-09-15

**Authors:** Robinson Karuga

**Affiliations:** 1 LVCT Health, Nairobi, Kenya

This is an interesting and timely article. I have a minor comment:

-  This last sentence in the results section seems misplaced and is hanging. Clarify whether it is supported by literature or whether this is the authors' opinion " Community empowerment in community dialogues tends towards health promotion activities."

Are the rationale for, and objectives of, the Systematic Review clearly stated?

Yes

Is the statistical analysis and its interpretation appropriate?

Not applicable

If this is a Living Systematic Review, is the ‘living’ method appropriate and is the search schedule clearly defined and justified? (‘Living Systematic Review’ or a variation of this term should be included in the title.)

Not applicable

Are sufficient details of the methods and analysis provided to allow replication by others?

Yes

Are the conclusions drawn adequately supported by the results presented in the review?

Yes

Reviewer Expertise:

Health Systems Research

I confirm that I have read this submission and believe that I have an appropriate level of expertise to confirm that it is of an acceptable scientific standard.

# Response

**Published:** 2024-09-16

**Authors:** Moriasi Nyanchoka

**Affiliations:** Health Economics Research Unit, KEMRI-Wellcome Trust Research Programme Nairobi, Nairobi, Nairobi County, Kenya

Thank you for reviewing our article. Please find our response to your comment below.

**Comment:** This last sentence in the results section seems misplaced and is hanging. Clarify whether it is supported by literature or whether this is the authors' opinion " Community empowerment in community dialogues tends towards health promotion activities."

**Response:** Thank you for pointing this out. This finding is supported by literature. We have added in-text citations.

# Reviewer report: Reviewer response for version 1

**DOI:** 10.21956/wellcomeopenres.25084.r95038

**Published:** 2024-09-06

**Authors:** Ramathebane Maseabata

**Affiliations:** 1 Department of Pharmacy, National University of Lesotho, Maseru, Lesotho

I reviewed this article, and here are my views about it. The article is rewritten, and there is an incorrect full-out of DHIS2, which is supposed to be the District Health Information System, not the Demographic Health Information System.

 Apart from this, I am happy with the article, and I recommend it for indexing.

Are the rationale for, and objectives of, the Systematic Review clearly stated?

Yes

Is the statistical analysis and its interpretation appropriate?

Yes

If this is a Living Systematic Review, is the ‘living’ method appropriate and is the search schedule clearly defined and justified? (‘Living Systematic Review’ or a variation of this term should be included in the title.)

Yes

Are sufficient details of the methods and analysis provided to allow replication by others?

Yes

Are the conclusions drawn adequately supported by the results presented in the review?

Yes

Reviewer Expertise:

Pharmacy Practice

I confirm that I have read this submission and believe that I have an appropriate level of expertise to confirm that it is of an acceptable scientific standard.

# Response

**Published:** 2024-09-16

**Authors:** Moriasi Nyanchoka

**Affiliations:** Health Economics Research Unit, KEMRI-Wellcome Trust Research Programme Nairobi, Nairobi, Nairobi County, Kenya

Thank you for reviewing our article. Please find our response to your comment below.

**Comment:** I reviewed this article, and here are my views about it. The article is rewritten, and there is an incorrect full-out of DHIS2, which is supposed to be the District Health Information System, not the Demographic Health Information System.

**Response:** We have corrected it. The sentence now reads “These report forms their respective catchment areas are then submitted to the supervisors, who aggregate the data in paper-based standardized MoH forms that are in turn submitted to the sub-national office (sub-county, county, district, or regional) for digital entry into the web-based national health information systems, the District Health Information System (DHIS2)”.
